# Illustrating the (in)visible: Understanding the impact of loss in adults living with secondary lymphedema after cancer

**DOI:** 10.3402/qhw.v9.24354

**Published:** 2014-08-21

**Authors:** Roanne Thomas, Ryan Hamilton

**Affiliations:** 1 Faculty of Health Science, University of Ottawa, Ottawa, ON, Canada; 2 Faculty of Arts, University of New Brunswick, Fredericton, NB, Canada

**Keywords:** Lymphedema, psychosocial, cancer, qualitative

## Abstract

Life with a disability is often riddled with paradoxes, one of which is being visibly marked, while personal experiences, losses, and challenges remain hidden. Our article draws attention to this paradox among people who live with secondary lymphedema after cancer (SLC). SLC is a relatively unfamiliar chronic condition within medical and lay discourses of cancer, which proves challenging for the many cancer survivors who are in search of information and understanding. Thirteen men and women with SLC were recruited from two research sites (Fredericton, NB, and Ottawa, ON, Canada) to participate in semi-structured interviews about the physical and psychosocial aspects of SLC. Using a methodology of interpretive description, our analysis of participant interviews reveals the complex ways in which men and women felt both visible and invisible within various contexts. We discuss three majors themes: (in)visibility and appearance related to material losses; (in)visibility and action connected to visible losses in function, as well as invisible struggles to care for oneself; and the loss of present and future well-being, as SLC renders some limitations visible while potentially obscuring a hopeful future indefinitely. Our research indicates that timely diagnosis of SLC would be an immediate first step in recognizing the physical and emotional dimensions of the condition. To accomplish this, increased awareness is needed. To enhance quality of life for those living with SLC, the development of new resources and psychosocial supports is also required.

Numerous scholars have written about the problems of being simultaneously visible (or hyper-visible) and invisible while a member of a marginalized group (Brueggemann, White, Dunn, Heifferon, & Cheu, 2001; Caldwell, 2010; Derby, 2012; Garland Thomson, 2005, 2006; Matthews, 2009). With secondary lymphedema after cancer (SLC), one is visibly marked by swelling (most commonly in the limbs), but also by the often bulky compression garments used to manage the condition. Discursively and socially, disability is understood as a deficiency (Thomas-MacLean & Miedema, 2005a), so while cancer survivors may be able to conceal the fact that they have had cancer, should they choose to do so, SLC is not as readily hidden. The prevailing cancer discourse means that the general public assumes that the disease is cured once treatments are complete and there is little awareness of long-term effects and treatments involved, such as the need to wear compression garments. At the same time, a dearth of information about lymphedema and a lack of awareness about this condition among health professionals both contribute to experiences of invisibility (Thomas-MacLean & Miedema, 2005a; Ryan et al., 2003). That is, patients’ symptoms often go unrecognized and untreated. Unlike other chronic illnesses, such as diabetes, or the very cancer that those with SLC have experienced, ignorance about SLC is pervasive (Shaw & Thomas, 2013, Shaw & Thomas, in press). All of this is compounded by an historical lack of regard for the psychosocial impact of lymphedema (Bogan, Powell, & Dudgeon, 2007; Fu et al., 2012).

There is, however, a growing body of literature documenting the psychosocial impact of SLC. Qualitative research shows that SLC results in changes to normalcy and one's sense of self (Thomas-MacLean, Miedema & Tatemichi, 2005b; Meiklejohn, Heesch, Janda, & Hayes, 2013). A recent systematic review of health-related quality of life outcomes for women with breast cancer-related lymphedema showed that the majority of studies reported poorer HRQOL outcomes in function and psychological well-being (Pusic et al., 2013). Another systematic review, addressing both upper and lower limb SLC, found statistically significant differences for social well-being (e.g., body image, sexuality) (Fu et al., 2012). The same review synthesizes qualitative findings from 12 studies, with the authors reporting that all studies described psychological distress and negative social impact (e.g., emotional disturbance, marginalization, isolation, and social abandonment) (Fu et al., 2012). Yet, despite visible markers of disability, such as compression garments, SLC remains somewhat invisible.

Scholars engaged with disability studies illustrate the multiple and intersecting ways in which visibility and invisibility might be understood. For instance, some disabilities are categorized as visible, while others are not. Mullins and Preyde (2013) explored the experiences of university students with invisible disabilities (i.e., those without a visible marker, such as the use of a wheelchair). While some students with dyslexia, attention deficit disorder, and mental illness indicated that a visual marker of disability might help with the removal of barriers to their education, most felt it was advantageous to be able to “pass” or to appear that they did not have disabilities, deciding when or if they would disclose. In another study, young female survivors of hemorrhagic stroke (considered to result in invisible disabilities) stated that culturally accepted visible markers of illness could mean that this type of disability would be taken more seriously (Stone, 2005). Yet, even with a highly visible marker of disability, people with disabilities encounter a lack of understanding about issues, such as social barriers, which renders some dimensions of experience invisible.

Drawing on several sources, Zitzelsberger (2010) demonstrates the ways in which seeing and visibility are socially equated with being known on some levels, but visibility from the perspective of people with disabilities is simultaneously an “imposed way of being seen” (Zitzelsberger, 2010, p. 400) which does not automatically result in being fully acknowledged. Others assert that “[d]isability … is couched in stares, whispers, pointing fingers” regarding the function or visible limitations of the body (Brueggeman et al., 2001, p. 387). Yet these authors, as Zitzelberger also alludes, note that visible disabilities are often invisible with respect to an understanding of the impact.

Noted disability scholar, Rosemarie Garland-Thompson problematizes visibility further. She states “Staring both registers and demands a response” (Garland Thompson, 2006, p. 174). She adds to theories of (in)visibility through her assertions that staring disrupts narratives, resulting in queries about ourselves and our possibilities. (In)visibility—in short, the paradoxical experience of being scrutinized while also having aspects of one's identity remain hidden—has also been explored in studies of race and sexuality in health care (e.g., Giddings & Smith, 2001; Mapedzahama, Rudge, West, & Perron, 2012), as well as the field of disability studies. Thus, from multiple perspectives, (in)visibility is highly relevant, both theoretically and empirically, to our research on SLC.

In this article, we attempt to make visible the impact of SLC, while describing the parallel problematic of visibility. The intent of our qualitative study was to explore experiences of loss and hope in men and women living with SLC. In the present analysis, loss is the broader theme in which (in)visibility resides. For instance, some participants reported material losses related to the fact that they were unable to wear jewellery or clothing they did prior to SLC. This loss remains invisible because of the lack of attention to the impact of SLC and what it means for peoples’ sense of identity. Similarly, while a negative impact on leisure and activity as a result of SLC has been explored in the literature (Miedema et al., 2008, Miedema et al., 2011), the impact of this loss is often rendered invisible.

## Methods

This research was guided by an interpretive description design, which aims to reveal “associations, relationships, patterns” and descriptions which will have a practical or clinical application (Thorne, Hislop, Armstrong, & Oglov, 2008). The descriptions emerging from our research have implications for nursing, the social sciences, and for rehabilitation. The experience of loss couched within the larger (in)visibility framework may provide avenues for psychosocial intervention.

Our project was based out of two research sites (Fredericton, NB, and Ottawa, ON). Thirteen participants were recruited through posters distributed at various physical (e.g., physiotherapy clinics) and web-based locations (e.g., newsletters of support groups) as well as through snowballing. Originally, we had planned to complete in-person interviews only. However, a few participants in the greater Ottawa area asked if they might be interviewed by telephone. Not wishing to exclude them, we applied for and received approval for an amendment to our original ethics application. Six participants were interviewed in person, and seven were interviewed by telephone. All interviews were audio recorded and transcribed verbatim.

### Participants

The inclusion criteria were men and women: a) who were 18 years of age or older; b) who had been diagnosed with upper or lower limb SLC for a minimum of 3 months prior to data collection; c) who were in the management phase of SLC treatment at the time of enrolment; d) were English speaking; and e) were willing to be audio recorded (for qualitative interviews).

The ages of the 13 participants (11 women, 2 men) ranged from 31 to 77. Eight of the 13 participants lived in urban areas. Five participants were working full-time. Participants had been diagnosed with cancer from 2 to 34 years (mean 10 years) prior to being interviewed. Six participants had breast cancer, four had melanoma, and three had reproductive cancers. Participants had been diagnosed with SLC from less than 1 year to 11 years post-cancer diagnoses (mean 8.5 years). Seven participants had lower limb SLC, while 6 were experiencing upper limb SLC. Only one participant had been hospitalized for SLC, but three participants had been prescribed antibiotics for infection. All participants provided written consent prior to participation in the study.

### Interviews

A brief questionnaire was administered at the beginning of the interviews in order to obtain data about socio-demographics, cancer and SLC diagnoses, and treatment. Next, our use of a semi-structured interview guide asked participants to discuss symptoms of SLC, its impact on various aspects of their lives (e.g., paid work, leisure, family). While topics were somewhat defined, they are influenced by other more open-ended guides used in other projects with people living with SLC and topics that those participants raised as significant (Thomas-MacLean & Miedema, 2005a; 2005b, Thomas-MacLean et al., 2009). Next, participants were asked how they conceptualized hope and what provided them with hope while living with SLC.

### Analytical approaches

Interpretive description draws on a number of methodological strands, including contemporary phenomenology (Thorne, 2008). Simply put, researchers working phenomenologically seek to understand the meaning underpinning everyday experiences (Bentz & Rehorick, 2008). While not purely phenomenological, our interpretation of the data centered on the meaning (i.e., loss and (in)visibility) of physiological and psychological symptoms, rather than their straightforward exposition. In this respect, our interpretive approach leans more heavily toward the phenomenological aspects of interpretive description than either grounded theory or ethnography, two other methodological antecedents associated with Thorne's methodology.

More pragmatically, discussion of emerging themes provided some direction for the analysis as the interviews were completed. More specifically, the research associates who completed the interviews met with the authors after each interview to identify issues with the interview guide and additional questions that might be asked. The research associates also highlighted key findings within the transcripts linked to the interviews they had completed (e.g., moments when participants became particularly emotional; ideas that participants themselves had highlighted as being important). As these tentative themes were identified, the lead authors had several discussions with the research associates and with each other in order to produce preliminary thematic categories. Prior to developing a tentative, coding framework within the software program, NVivo 10, all transcripts were printed. Handwritten memos and text highlights helped the authors generate the coding framework in NVivo, then, all of the interview transcripts were coded line by line by one of the authors after modifications to the framework (i.e., the initial thematic categories). The interpretations of the data and thematic categories were then further discussed, refined, and synthesized by the authors.

Following the initial line-by-line coding and team discussions, the resulting nodes or thematic categories were subsequently collapsed to generate a broader exposition of the central themes. At this point, the data and associated interpretations were queried for patterns related specifically to (in)visibility (i.e., data were subject to “transformation” and “abstraction”) (Thorne, 2008, p. 164).

### Ethical considerations

The study was approved by Research Ethics Boards at the University of Ottawa and the University of New Brunswick (UNB—REB #2012-063, Ottawa—#H05-12-04). The participants were informed both verbally and in writing about the study objectives and procedures. Written, informed consent was obtained from those who participated.

## Results

Herein, we explore three key themes emerging from our interpretation of in-depth interview data. (In)visibility is woven throughout each of the themes. The first theme, (in)visibility and appearance, relates to living with a swollen limb and material losses which make individuals with SLC highly visible, while they live with a largely invisible or unknown condition. The second theme, (in)visibility and action, is connected to function and leisure (or visible losses), but also a limited ability to play an active role in self-care because of the lack of recognition (invisibility) of this condition. The final, more abstract theme, invites consideration of loss of the present, because individuals may be unable to completely forget they have had cancer due to the presence of SLC. They are also aware that the condition is permanent and may worsen in the future, making uncertainty more visible as a potentially cancer-free future is also lost or rendered invisible.

### (In)visibility and appearance

The first theme, (in)visibility and appearance, relates to living with a swollen limb and material losses which make individuals with SLC highly visible, while they live with a largely invisible or unknown condition. Participants experienced the loss of their ability to present themselves as visibly healthy, or unmarked by an illness such as cancer. One participant said:

I guess the compression bandages help, and I know I can wrap my hands, but even that is really unattractive so I don't wear it often. It is so hot wearing a compression sleeve and it just draws more attention to my arm that if it was swollen. I don't like bringing attention to the fact that I had cancer. I just want to put it behind me, so this sleeve isn't really something I wear unless I feel I really have to. (P022013, FEM, ARM)

A woman with lower limb SLC expressed similar feelings: “… Sometimes I feel very self-conscious when I'm out in public because it is fairly visible and that's kind of annoying in a way” (P082311, FEM, LEG). Swollen limbs and compression garments mean that SLC is highly “visible,” even to strangers, but with a general lack of SLC awareness, one woman commented that: “It was easier to say I was in a car accident than explain lymphedema” (P121910, FEM, ARM) to people who asked her what happened to her arm.

The anticipated visibility of SLC may also result in an almost continual consideration of clothing as this participant demonstrates when she talked about getting dressed in the morning:

… It's not that I'm that fixated on it, but I think, you know, women like to look nice in the morning when they get ready and it's constantly me adjusting to the lymphedema leg and figuring out what's gonna work. (P080901, FEM, LEG).

Similarly, another participant spoke about “hiding” her leg under long pants or skirts (P101007, FEM, LEG). A male participant also spoke about concealing his compression garment:

I typically wear long pants and so on and so forth. Ah, when everyone around me is dressed for summer, that's a little bit frustrating, and sometimes I'm a little bit jealous, and I know that nobody is forcing me to do what I do. (P091404, MALE, LEG)

The constraints and limits of SLC are visible in their materiality or clothing selection.

Concealed compression garments or the lack of visible markers of illness also result in other challenges as the condition becomes invisible. One participant noted that she had a disabled parking permit as walking could be difficult with leg SLC. She said:

People look at you as if to say, “Well you're perfectly healthy, what the hell's wrong with you.” [Interviewer: It's not visible.] That's right. And, I've actually been yelled at by one older gentleman saying that I was taking up the space of somebody that would really need it and I said “Oh, did you have cancer twice and then end up with lymphedema?” “No.” I said, “Well I did” and walked off. So, I hope it had some effect on him because I was mad because he was hooting and hollering. I was also humiliated because he was yelling and everybody in the parking lot was looking at me. (P101007, FEM, LEG)

This potential visibility of compression garments, coupled with the lack of visibility or awareness of the condition creates a burden of explanation that has psychosocial implications. While the material aspects of SLC may be visible, the impact on emotional well-being is often hidden.

Similarly, other material (tangible) losses may be visible to participants and their friends/families, with the impact on identity being more invisible. One participant commented repeatedly on her inability to wear jewellery because of upper limb SLC. She said: “So, I'm very selfish here because I've been wanting to wear my pretty jewellery …. That's kind of my aim, kind of ridiculous, but that's who I am” (P090702, FEM, ARM). The inability to wear jewellery may represent a visible loss to those who know her, but it is not clear if family members or friends appreciate the significance of jewellery to identity or “who I am.” Participants’ words direct attention to visible or material losses, but also how these losses signify and shape the emotional impact which is often invisible. This tension is also present in the second theme of our research on loss and SLC.

### (In)visibility and action

Participants’ abilities to be active are limited by SLC in two key ways. The first relates to function. All of the participants talked about limitations to activity (e.g., leisure activities, paid work) or function. For most, these losses were more profound as they involved activities of daily living, while for a few participants, losses were only experienced intermittently or were anticipated if participants’ symptoms worsened. The second aspect of loss related to activity is less visible and is connected to the inability to play an active role in self-care because of the limited knowledge of health professionals. Although the two sub-themes may seem somewhat disconnected, both leave participants limited in their abilities to care for themselves physically, and both are connected to emotional losses or impacts that may be invisible.

In speaking of losses associated with everyday activities, one woman said:

I'm a spinner so I use my right foot a lot and I also have an electric wheel which helps me when I'm spinning because once you start doing that it is like an hour and half straight so I couldn't do it manually. So I have an electric wheel for that. But … and I'm supposed to keep my leg up as much as possible, so yeah, that kind of interferes with everything. (P082311, FEM, LEG)

For this participant, leg SLC interferes with her livelihood, which is also her craft—both are connected to identity.

Another participant with leg SLC spoke about the impact of leg SLC on leisure. She talked about taking her first trip since being treated for SLC. She relied on friends who accompanied her to assist with putting her stockings on because back surgery had resulted in less flexibility. She said that she felt it was reasonable to ask her friends for this support with her garments for a short trip (3 days), but did not feel that she would be comfortable asking for this assistance on a longer trip, saying: “It's asking a lot.” Her ability to travel was therefore limited by SLC and her reluctance to ask for extensive support (P091905, FEM, LEG).

Other participants with leg SLC spoke of challenges posed in activities such as horseback riding, golf, and cycling, while a woman with arm SLC who lives in a rural area spoke of experiencing limitations to outdoor activities:

I love to rake and I love to cut the grass. I love to pile rocks and I love to be very, very physical but I can't be repetitive with my ‘stupid lymphedema arm’, which I call it, when I am doing that type of stuff. It drives me nuts and I take lots of breaks. And, before, I could get the work done in two weeks, instead of three months …. But, by time I get it all done now, it is Fall …. That annoys me because I love to be outside, but I am learning to forgive myself and get more help when I can and just live with it. (P121910, FEM, ARM)

For all participants, the physical impact on function was immediately visible and readily described. However, the more invisible emotional impact of these limitations was underpinning many of their discussions of these physical losses as the last quotation demonstrates through the use of phrases such as “It drives me nuts” and “I am learning to forgive myself.” Many of the participants said they were “frustrated” by their limitations. One woman stated:

“I have to get my husband to sort of do things because I don't have enough strength, whether it's sunscreen lids, or opening a box. I get very frustrated because I can't do what I used to be able to do because I don't have the strength now” (P190906, FEM, ARM).

Also frustrating was the overall invisibility of SLC as a chronic illness which limited participants’ abilities to play an active role in self-care. Most participants spoke of the lack of knowledge of health professionals and having to rely on the Internet for information. One man said that he had to search for treatment providers and information independently and that he had no assistance from health professionals: “I had to figure things out on my own …. It's very frustrating … I didn't receive any support in this area” (P091404, MALE, LEG). This participant went on to say that his reason for participating in our study was to raise awareness of the lack of information and support for SLC.

A female participant described her search for information and treatment after experiencing swelling in her leg. She approached her physician who was concerned about a cancer recurrence. After waiting for a few months for tests and their results, this participant started to search for information about leg swelling after cancer:

… up came all these websites about lymphedema. And I had never heard of the word before, I didn't know how to spell it, I didn't know how to pronounce it, I had never ever seen that word. As I searched through all the sites, I looked and thought “This is it. This is what I've got.” So when I went to my family doctor, she said “Well, you know you really have to be careful about what you see on the internet.” I said, “I know that. I'm an educated person! Look at these websites—they're Stanford University, the Mayo Clinic ….” And I said, “Look I've got nine out of the ten risk factors and criteria. I'm sure this is what it is.” I had found out that there was a lymphedema clinic at [hospital]. I gave my family doctor that number and said, “I need a referral. Could you please get me in as soon as possible?” Ironically, that lymphedema clinic is two doors down from the surgical oncologist I had been seeing for the last five years during follow-up to my cancer surgery. (P080901, FEM, LEG)

This participant's diagnostic narrative was shared by several other participants. Physicians were not generally well-informed about treatment options either. One participant said that her surgeon said that she had SLC and “there's not much you can do about it” (P091905, FEM, LEG). She and her daughter searched for information, located a SLC care provider and arranged for participation in her province's assistive devices program in order to cover some of the costs associated with her compression garments.^1^

Another participant said that she found support from a physiotherapist, but

The doctors are very, um, I don't know if they're unhelpful or ignorant but they don't regard lymphedema as anything which requires treatment. It's a cosmetic thing from their point of view, not something which needs to be treated. (P091906, FEM, ARM).

Although participants with upper limb SLC described the invisibility of their condition in the health care system, participants with SLC in their lower limbs felt that arm SLC was more recognized or visible than their own experiences. This sentiment is supported by the fact that some SLC programs in Canada are funded by breast cancer organizations, so they are limited to women with arm morbidity. A participant with leg SLC said:

“It's a problem for other cancer survivors and not just people who have survived breast cancer, and you know, there is not just lymphedema of the arm. It's in other body parts” (P082311, FEM, LEG).

Thus, as with the first theme, the psychosocial impact of being unable to take an active role in various aspects of life is largely invisible.

### (In)visibility of the present and future

The final, more abstract theme, invites consideration of loss of the present because individuals may be unable to engage in activities spontaneously, in ways they had taken for granted in the past, and while SLC symptoms are visible, the effect is not so apparent. To illustrate, one participant said:

Well, I can't go swimming at the drop of a hat …. You know, people say, “Oh, let's go out for a bike ride.” I have problems with that. Or, you know, “Let's go to the water park.” I can't do that because I can't be without my stockings all day. (P101008, FEM, LEG)

Limits on spontaneity may signify the loss of some potential to live fully in the present, without restrictions or without having to be future-directed.

Some participants said that SLC meant that they were unable to completely forget that they had cancer due to the presence of SLC which also affected their ability to live in a previously taken for granted present. Participants also spoke of becoming aware that the condition is permanent and both the present and future have now been altered. One participant mentioned that this was also an issue for the people around her:

“I think what is startling for people are when they are shocked that my lymphedema is not going to go away …. It is a lifetime condition. That scares a lot of friends and family” (P121910, FEM, ARM).

The future of wellness becomes invisible, while the visible future now includes a disabling illness.

Also influencing the present is an awareness that SLC may worsen in the future:

“… there is at least some fear that if I injure the affected limb that maybe I could get an infection and have problems” (P091404, MALE, LEG).

Through monitoring their swelling, participants continually make illness visible to themselves which may extend to a hypervigilance:

‘‘And then it's always no matter what you do, be careful you don't make it worse … I guess I just hope it doesn't get worse …. You know, I'm 54 and I think ‘What am I going to be like when I'm 70?’” (P101008, FEM, LEG).

Unlike cancer, where one might be able to occasionally forget about the possibility of a recurrence, SLC is permanent. The demands of self-management are constant reminders of that permanence, marking a loss and rendering a healthy present and future life somewhat invisible.

## Discussion

SLC presents unique challenges to cancer survivors. Changes in appearance and compression garments leave people with SLC marked by two illnesses, or a type of visibility that many participants indicated was problematic, even while they might wish to forget for a time that they had cancer or that they are living with lymphedema. Losses related to a “normal” appearance may be quite visible, while other social barriers and challenges are related to the invisibility of a disabling condition. However, the emotional impact is not often discussed or acknowledged and the connections of identity to material (visible and tangible) losses are often underexplored or invisible. Our findings therefore support those of Fu et al. (2012) and Pusic et al. (2013) who have documented negative social impacts of SLC, as well as psychological distress. Yet, the losses associated with such impacts are not often understood. Conveying their meanings, or making them more visible, may assist health professionals and others to better understand the impact of SLC on the everyday and to enhance support accordingly.

In the face of having survived cancer, being unable to wear jewellery or find clothing that fits properly and looks attractive, could seem trivial. This may account for some of the minimizing of symptoms by some participants. However, clothing and jewellery can be central to one's identity and are intrinsic to one's routines. Jewellery, in particular, can be evocative of memories and may convey one's personal style, or may be an expression of creativity. Being unable to wear what one used to do is also a reminder that life has been altered and the anticipated future is no longer what it once was. For people with SLC, normalcy shifts and our participants demonstrate changes to their sense of self as Meiklejohn et al. (2013) and Thomas-MacLean et al., (2009) have also noted.

Further, and by extension, the loss of leisure does not simply result in the inability to perform physical tasks, but may also result in changes to self-perception. While the negative impact of SLC on leisure has been documented (Miedema et al., 2008), such losses have not been described with respect to identity shifts. Individuals with SLC may also become invisible to their social networks or people with whom they golfed, quilted, or walked for example. This means valuable social support may also be lost, resulting in increased invisibility among peers. While social barriers have been identified in the disability literature (Mullins & Preyde, 2013; Stone, 2005), the loss of a network built through leisure activities has not been explored in the body of work outlining the psychosocial impact of SLC. In this case, and for our participants, the meaning of disability is not seen, therefore it is not known, following Zitzelsberger's (2010) line of reasoning.

Despite its impact, there remains a lack of SLC awareness among many health professionals, already noted over 10 years ago (Thomas-MacLean, Miedema & Tatemichi, 2005b; Ryan et al., 2003). This lack of awareness left participants somewhat powerless to actively engage in care. They were also unable to express their frustrations and other emotions. Diagnosis would be an immediate first step in recognizing the physical and emotional dimensions of SLC, but a lack of attention to diagnosis meant that even this essential first step in the clinical pathway was overlooked or invisible.

Complicating the invisibility of the psychosocial impact and limitations on activity, is the awareness of the permanence of the condition, a topic rarely, if ever, explored in existing SLC literature. Both survivors and family members may expect that, after surviving cancer, individuals will move from fear and depression to hope or a return to normalcy. SLC complicates this because one does not resume life as normal, but is left to cope with a chronic, incurable and often visible condition. As our participants demonstrate, this leaves them vulnerable to staring or an imposition of sight that does not necessarily result in understanding (Zitzelsberger, 2010).

Simultaneously, there may be expectations that negative emotions will dissipate or become invisible as patients move into a survivorship phase of cancer. People with SLC are then left alone to address their fears about worsening symptoms and to comfort friends and family members who may also be frightened by the condition's permanence and the loss of an anticipated, healthy present. This overlapping of two illnesses and their associated existential dilemmas are not well-recognized in the bodies of literature on the psychosocial impact of cancer and SLC which tend to be housed in separate silos. That is, fears of cancer recurrence are addressed and, limitedly, the psychosocial impact of SLC has been explored, but the two domains have not been investigated jointly. To the best of our knowledge, in research and in practice, they remain separate, and the overlap is invisible, although cancer and SLC were not experienced as such by our participants.

### Methodological considerations

Our study was well-balanced with respect to the proportion of participants (roughly half) living with upper or lower limb SLC. Another strength of our study, also related to our sample, is the representation of diversity in age and time since cancer and SLC diagnoses, suggesting that a wide range of experiences was captured. However, despite our best attempts to enroll men to the study, only two of the 13 participants were male. These participants appeared to have much in common with the women, but the lack of men may be viewed as a limitation.

Further strengthening our study were efforts undertaken to share our findings, including quotations from participants, with a different group of participants in a follow-up study of a psychosocial intervention for people living with SLC after cancer. These participants verified that the themes of the present project were congruent with their experiences, providing an indication of the trustworthiness of the data. One more strength of our study is that the report was also shared with two SLC experts—one a patient who was a participant in the present study, also a leading advocate for lymphedema care in Canada, and the other a physician whose lymphedema clinic is internationally recognized. Both agreed that the findings are reflective of their experiences and illustrative of their work (e.g., consistent with inquiries received to a provincial help line).

While researchers are beginning to explore the psychosocial impact of SLC, this work is original because loss has not been explicitly explored. Also, to the best of our knowledge, the concept of (in)visibility, while examined in relation to other marginalized populations, has not been connected to experiences of SLC.

## Conclusion

Study participants demonstrate both the invisibility and visibility of the impact of SLC, as well as the types of losses they experienced as a result. Opportunities (i.e., community-based workshops and other interventions) to make this condition and the associated emotions visible could be highly beneficial to cancer survivors with SLC, especially since there are currently few resources available to assist with the psychosocial adjustments necessary to living well despite various limitations and constraints. Our research demonstrates important directions for empathic care, but also for setting priorities for psychosocial support and cancer rehabilitation more broadly. In-depth knowledge of SLC could also support the actual development of new and more holistic initiatives in cancer rehabilitation.

## Footnotes

1 It should be noted that not all Canadian provinces provide coverage through public health insurance for compression garments (e.g., at the time of data collection, Ontario did, but Quebec did not). While Canada has a national health care system, it is administered provincially, which accounts for some of the disparities in coverage.
